# Crystal structure of 2-benzyl­amino-4-*p*-tolyl-6,7-di­hydro-5*H*-cyclo­penta­[*b*]pyridine-3-carbo­nitrile

**DOI:** 10.1107/S2056989015000572

**Published:** 2015-01-21

**Authors:** R. A. Nagalakshmi, J. Suresh, S. Maharani, R. Ranjith Kumar, P. L. Nilantha Lakshman

**Affiliations:** a Department of Physics, The Madura College, Madurai 625 011, India; b Department of Organic Chemistry, School of Chemistry, Madurai Kamaraj University, Madurai 625 021, India; c Department of Food Science and Technology, University of Ruhuna, Mapalana, Kamburupitiya 81100, Sri Lanka

**Keywords:** crystal structure, cyclo­penta­[*b*]pyridine, 2-amino-3-cyano­pyridine, pyridine-3-carbo­nitrile, hydrogen bonding, C—H⋯π inter­actions, π–π inter­actions

## Abstract

The title compound, C_23_H_21_N_3_, comprises a 2-amino-3-cyano­pyridine ring fused with a cyclo­pentane ring. The later adopts an envelope conformation with the central methyl­ene C atom as the flap. The benzyl and and *p*-tolyl rings are inclined to one another by 56.18 (15)°, and to the pyridine ring by 81.87 (14) and 47.60 (11)°, respectively. In the crystal, mol­ecules are linked by pairs of N—H⋯N_nitrile_ hydrogen bonds, forming inversion dimers with an *R*_2_^2^(12) ring motif. The dimers are linked by C—H⋯π and π–π inter­actions [centroid–centroid distance = 3.7211 (12) Å], forming a three-dimensional framework.

## Abstract 2

In the crystal of the title compound, mol­ecules are linked by pairs of N—H⋯N_nitrile_ hydrogen bonds, forming inversion dimers with an 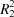
(12) ring motif. The dimers are linked by C—H⋯π and π–π inter­actions [centroid–centroid distance = 3.7211 (12) Å], forming a three-dimensional framework.

## Chemical context   

The pyridine nucleus is prevalent in numerous natural products and is extremely important in the chemistry of biological systems (Bringmann *et al.*, 2004 ▸). Many naturally occurring and synthetic compounds containing the pyridine scaffold possess inter­esting pharmacological properties (Temple *et al.*, 1992 ▸). Among them, 2-amino-3-cyano­pyridines have been identified as IKK-β inhibitors (Murata *et al.*, 2003 ▸). The above observations prompted us to synthesize the title compound, which contains a pyridine 3-carbo­nitrile group, and we report herein on its crystal structure. 
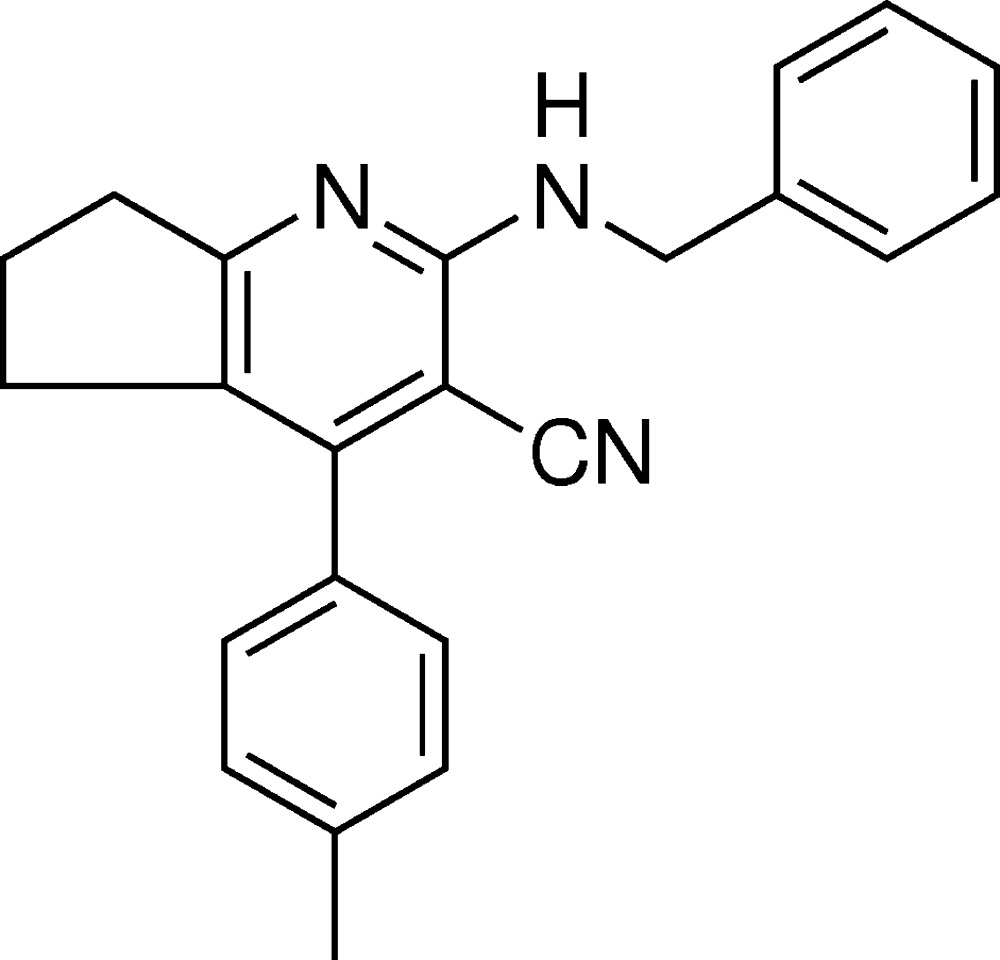


## Structural commentary   

**Figure 1:**
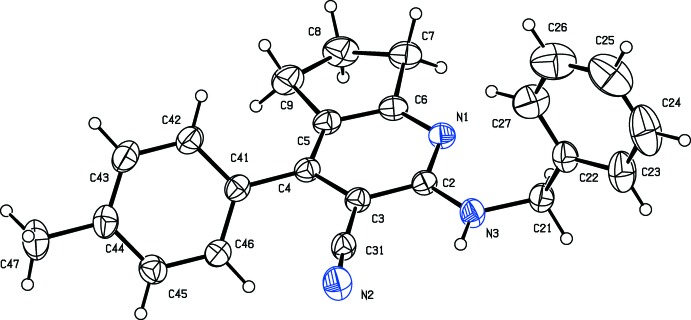
The mol­ecular structure of the title compound, showing the atom labelling. Displacement ellipsoids are drawn at the 30% probability level.

The mol­ecular structure of the title compound is shown Fig. 1 ▸. As expected, the pyridine ring (N1/C2–C6) is almost planar (r.m.s. deviation = 0.009 Å). The cyclo­pentane ring fused with the pyridine ring adopts an envelope conformation with atom C8 as the flap, deviating by 0.3505 (1)Å from the mean plane defined by atoms (C5/C6/C7/C9). In the CH_2_–NH_2_ chain, the C—N bond lengths [C2—N3 = 1.349 (3) and N3—C21 = 1.437 (3) Å] are comparable with those reported for a similar structure (Nagalakshmi *et al.*, 2014 ▸). The endocyclic angle at C5 is contracted to 118.73 (19)° while that at C6 is expanded to 126.2 (2)°, due to the fusion of the five- and six-membered rings. Steric hindrance rotates the benzyl ring (C22–C27) out of the plane of the central pyridine ring by 81.87 (14)°. This twist may be due to the non-bonded inter­actions between one of the *ortho-*H atoms of the benzene ring and atom H21*B* of the CH_2_–NH_2_ chain. The benzyl and and *p*-tolyl (C41–C46) rings are inclined to one another by 56.18 (15)°, while the *p*-tolyl ring is inclined to the pyridine ring by 47.60 (11)°.

## Supra­molecular features   

**Figure 2:**
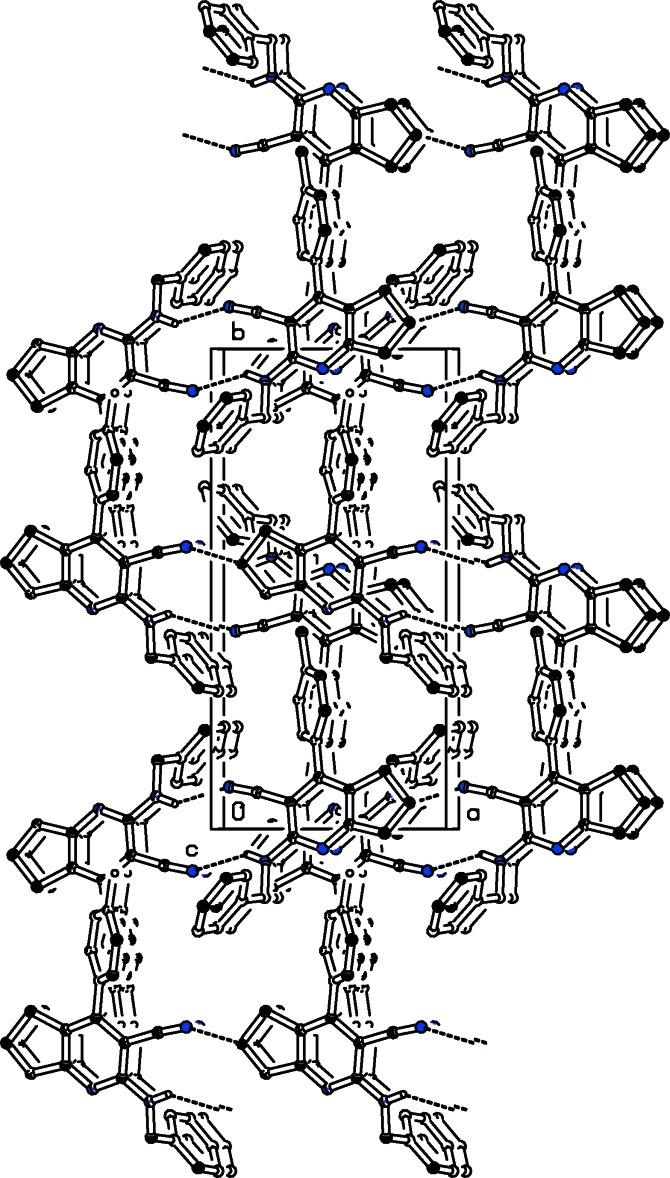
A view along the *c* axis of the crystal packing of the title compound. Hydrogen bonds are shown as dashed lines (see Table 1 ▸ for details) and H atoms not involved in hydrogen bonding have been omitted for clarity.

In the crystal, mol­ecules are linked *via* pairs of N—H⋯N_nitrile_ inter­actions, forming inversion dimers which enclose 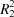
(12) ring motifs. The dimers are connected through weak C—H⋯π inter­actions involving the CN group as acceptor (Table 1 ▸ and Fig. 2 ▸). They are further connected by slipped parallel π–π stacking inter­actions involving the pyridine rings of inversion-related mol­ecules [*Cg*1⋯*Cg*1^i^ = 3.7211 (12), normal distance = 3.5991 (8), slippage = 0.945 Å; *Cg*1 is the centroid of the N1/C2–C6 ring; symmetry code: (i) −*x* + 1, −*y*, −*z*], resulting in the formation of a three-dimensional framework.

## Database survey   

Similar structures reported in the literature include 2-[2-(4-chloro­phen­yl)-2-oxoeth­oxy]-6,7-di­hydro-5*H*-cyclo­penta­[*b*]pyridine-3-carbo­nitrile (Mazina *et al.*, 2005 ▸) and 2-benzylamino-4-(4-meth­oxy­phen­yl)-6,7,8,9-tetra­hydro-5*H*-cyclohepta[*b*]pyridine-3-carbo­nitrile (Nagalakshmi *et al.*, 2014 ▸). In the first compound, the fused cyclo­pentane ring has an envelope conformation with the central methyl­ene C atom as the flap, similar to the situation in the title compound.

## Synthesis and crystallization   

A mixture of cyclo­penta­none (1 mmol) 1, 4-methyl­benzaldehyde (1 mmol), malono­nitrile (1 mmol) and benzyl­amine were taken in ethanol (10 mL) to which *p*-toluene­sulfonic acid (*p*-TSA) (1 mmol) was added. The reaction mixture was heated under reflux for 2–3 h. The reaction progress was monitored by thin layer chromatography. After completion of the reaction, the mixture was poured into crushed ice and extracted with ethyl acetate. The excess solvent was removed under vacuum and the residue was subjected to column chromatography using a petroleum ether/ethyl acetate mixture (97:3 *v*/*v*) as eluent to obtain the pure product. The product was recrystallized from ethyl acetate, affording colourless crystals of the title compound (yield: 70%, m.p.: 434 K).

## Refinement   

Crystal data, data collection and structure refinement details are summarized in Table 2 ▸. The NH and C-bound H atoms were placed in calculated positions and allowed to ride on their carrier atoms: N—H = 0.86 Å, C—H = 0.93–0.97 Å, with U_iso_(H) = 1.5U_eq_(C) for methyl H atoms and = 1.2U_eq_(N,C) for other H atoms.

## Supplementary Material

Crystal structure: contains datablock(s) global, I. DOI: 10.1107/S2056989015000572/su5061sup1.cif

Structure factors: contains datablock(s) I. DOI: 10.1107/S2056989015000572/su5061Isup2.hkl

Supporting information file. DOI: 10.1107/S2056989015000572/su5061Isup3.cml

CCDC reference: 1042906

Additional supporting information:  crystallographic information; 3D view; checkCIF report

## Figures and Tables

**Table 1:** Hydrogen-bond geometry (, ) *Cg*1 is the centroid of the N1/C2C6 pyridine ring.

*D*H*A*	*D*H	H*A*	*D* *A*	*D*H*A*
N3H3N2^i^	0.86	2.25	2.982(3)	144
C47H47*A* *Cg*1^ii^	0.96	2.84	3.681(4)	147

Symmetry codes: (i) 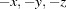; (ii) 
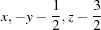
.

**Table 2:** Experimental details

Crystal data	
Chemical formula	C_23_H_21_N_3_
*M*_r_	339.43
Crystal system, space group	Monoclinic, *P*2_1_/*c*
Temperature (K)	293
*a*, *b*, *c* ()	8.6826(4), 17.7282(9), 12.0400(6)
()	94.253(2)
*V* (^3^)	1848.18(16)
*Z*	4
Radiation type	Mo *K*
(mm^1^)	0.07
Crystal size (mm)	0.21 0.19 0.18

Data collection	
Diffractometer	Bruker Kappa APEXII
Absorption correction	Multi-scan (*SADABS*; Bruker, 2004 ▸)
*T*_min_, *T*_max_	0.967, 0.974
No. of measured, independent and observed [*I* > 2(*I*)] reflections	29178, 3452, 2262
*R*_int_	0.034
(sin /)_max_ (^1^)	0.606

Refinement	
*R*[*F* ^2^ > 2(*F* ^2^)], *wR*(*F* ^2^), *S*	0.058, 0.192, 1.08
No. of reflections	3452
No. of parameters	237
No. of restraints	1
H-atom treatment	H-atom parameters constrained
_max_, _min_ (e ^3^)	0.29, 0.21

Computer programs: *APEX2* and *SAINT* (Bruker, 2004 ▸), *SHELXS2013* (Sheldrick, 2008 ▸), *SHELXL2014* (Sheldrick, 2015 ▸) and *PLATON* (Spek, 2009 ▸).

## supplementary crystallographic information

### Crystal data

**Table:**

C_23_H_21_N_3_	*F*(000) = 720
*M**_r_* = 339.43	*D*_x_ = 1.220 Mg m^−^^3^
Monoclinic, *P*2_1_/*c*	Mo *K*α radiation, λ = 0.71073 Å
*a* = 8.6826 (4) Å	Cell parameters from 2000 reflections
*b* = 17.7282 (9) Å	θ = 2–31°
*c* = 12.0400 (6) Å	µ = 0.07 mm^−^^1^
β = 94.253 (2)°	*T* = 293 K
*V* = 1848.18 (16) Å^3^	Block, colourless
*Z* = 4	0.21 × 0.19 × 0.18 mm

### Data collection

**Table:**

Bruker Kappa APEXII diffractometer	2262 reflections with *I* > 2σ(*I*)
Radiation source: fine-focus sealed tube	*R*_int_ = 0.034
ω and φ scans	θ_max_ = 25.5°, θ_min_ = 2.1°
Absorption correction: multi-scan (*SADABS*; Bruker, 2004)	*h* = −10→9
*T*_min_ = 0.967, *T*_max_ = 0.974	*k* = −21→21
29178 measured reflections	*l* = −14→14
3452 independent reflections	

### Refinement

**Table:**

Refinement on *F*^2^	Hydrogen site location: inferred from neighbouring sites
Least-squares matrix: full	H-atom parameters constrained
*R*[*F*^2^ > 2σ(*F*^2^)] = 0.058	*w* = 1/[σ^2^(*F*_o_^2^) + (0.1007*P*)^2^ + 0.5115*P*] where *P* = (*F*_o_^2^ + 2*F*_c_^2^)/3
*wR*(*F*^2^) = 0.192	(Δ/σ)_max_ < 0.001
*S* = 1.08	Δρ_max_ = 0.29 e Å^−^^3^
3452 reflections	Δρ_min_ = −0.21 e Å^−^^3^
237 parameters	Extinction correction: *SHELXL2014* (Sheldrick, 2015), Fc^*^=kFc[1+0.001xFc^2^λ^3^/sin(2θ)]^-1/4^
1 restraint	Extinction coefficient: 0.017 (4)

### Special details

**Table:**

Geometry. All e.s.d.'s (except the e.s.d. in the dihedral angle between two l.s. planes) are estimated using the full covariance matrix. The cell e.s.d.'s are taken into account individually in the estimation of e.s.d.'s in distances, angles and torsion angles; correlations between e.s.d.'s in cell parameters are only used when they are defined by crystal symmetry. An approximate (isotropic) treatment of cell e.s.d.'s is used for estimating e.s.d.'s involving l.s. planes.

### Fractional atomic coordinates and isotropic or equivalent isotropic displacement parameters (Å^2^)

**Table:**

	*x*	*y*	*z*	*U*_iso_*/*U*_eq_	
C2	0.3577 (2)	−0.01812 (12)	0.13085 (17)	0.0430 (5)	
C3	0.3439 (2)	0.05541 (12)	0.08637 (17)	0.0442 (5)	
C4	0.4663 (2)	0.10693 (12)	0.09975 (17)	0.0440 (5)	
C5	0.5989 (2)	0.08141 (13)	0.15993 (17)	0.0485 (6)	
C6	0.6012 (2)	0.00880 (13)	0.20121 (18)	0.0484 (6)	
C7	0.7526 (3)	−0.00857 (16)	0.2642 (2)	0.0644 (7)	
H7A	0.7425	−0.0086	0.3439	0.077*	
H7B	0.7922	−0.0571	0.2424	0.077*	
C8	0.8547 (3)	0.05436 (17)	0.2313 (2)	0.0738 (8)	
H8A	0.9171	0.0380	0.1722	0.089*	
H8B	0.9231	0.0700	0.2945	0.089*	
C9	0.7499 (3)	0.11988 (16)	0.1912 (2)	0.0676 (7)	
H9A	0.7397	0.1565	0.2501	0.081*	
H9B	0.7892	0.1450	0.1275	0.081*	
C21	0.2359 (3)	−0.14216 (13)	0.1612 (2)	0.0534 (6)	
H21A	0.3410	−0.1584	0.1812	0.064*	
H21B	0.1921	−0.1762	0.1042	0.064*	
C22	0.1449 (3)	−0.14823 (13)	0.2613 (2)	0.0549 (6)	
C23	0.0421 (3)	−0.20537 (17)	0.2721 (3)	0.0822 (9)	
H23	0.0233	−0.2399	0.2145	0.099*	
C24	−0.0354 (4)	−0.2124 (2)	0.3698 (4)	0.1126 (14)	
H24	−0.1048	−0.2517	0.3776	0.135*	
C25	−0.0081 (5)	−0.1610 (3)	0.4533 (4)	0.1160 (14)	
H25	−0.0573	−0.1661	0.5189	0.139*	
C26	0.0889 (4)	−0.1031 (3)	0.4417 (3)	0.1090 (12)	
H26	0.1041	−0.0673	0.4979	0.131*	
C27	0.1653 (3)	−0.0970 (2)	0.3468 (3)	0.0825 (9)	
H27	0.2330	−0.0569	0.3399	0.099*	
C31	0.1989 (3)	0.07613 (12)	0.03276 (19)	0.0491 (5)	
C41	0.4499 (2)	0.18393 (12)	0.05337 (18)	0.0463 (5)	
C42	0.4905 (3)	0.24647 (14)	0.1181 (2)	0.0582 (6)	
H42	0.5339	0.2396	0.1904	0.070*	
C43	0.4680 (3)	0.31822 (14)	0.0777 (2)	0.0665 (7)	
H43	0.4938	0.3591	0.1237	0.080*	
C44	0.4076 (3)	0.33109 (14)	−0.0303 (2)	0.0607 (7)	
C45	0.3693 (3)	0.26900 (14)	−0.0952 (2)	0.0579 (6)	
H45	0.3290	0.2760	−0.1682	0.069*	
C46	0.3891 (3)	0.19646 (13)	−0.05444 (19)	0.0507 (6)	
H46	0.3613	0.1556	−0.1001	0.061*	
C47	0.3824 (4)	0.40937 (16)	−0.0747 (3)	0.0918 (10)	
H47A	0.3738	0.4080	−0.1546	0.138*	
H47B	0.4681	0.4407	−0.0493	0.138*	
H47C	0.2890	0.4296	−0.0485	0.138*	
N1	0.4863 (2)	−0.04087 (10)	0.19013 (15)	0.0480 (5)	
N2	0.0782 (2)	0.08761 (13)	−0.0074 (2)	0.0701 (6)	
N3	0.2394 (2)	−0.06733 (10)	0.11551 (16)	0.0546 (5)	
H3	0.1593	−0.0527	0.0750	0.066*	

### Atomic displacement parameters (Å^2^)

**Table:**

	*U*^11^	*U*^22^	*U*^33^	*U*^12^	*U*^13^	*U*^23^
C2	0.0386 (11)	0.0454 (12)	0.0450 (12)	−0.0015 (9)	0.0021 (9)	−0.0008 (9)
C3	0.0394 (10)	0.0474 (12)	0.0457 (11)	−0.0040 (9)	0.0021 (8)	−0.0005 (9)
C4	0.0408 (11)	0.0484 (12)	0.0429 (11)	−0.0043 (9)	0.0029 (9)	−0.0027 (9)
C5	0.0390 (12)	0.0574 (14)	0.0487 (12)	−0.0073 (10)	0.0004 (9)	−0.0039 (10)
C6	0.0396 (11)	0.0586 (14)	0.0466 (12)	0.0012 (10)	0.0000 (9)	−0.0032 (10)
C7	0.0475 (13)	0.0766 (17)	0.0669 (16)	0.0002 (12)	−0.0104 (11)	0.0000 (13)
C8	0.0439 (14)	0.099 (2)	0.0761 (18)	−0.0074 (14)	−0.0084 (12)	0.0033 (15)
C9	0.0488 (14)	0.0777 (18)	0.0745 (17)	−0.0163 (13)	−0.0067 (12)	−0.0030 (13)
C21	0.0517 (13)	0.0445 (13)	0.0634 (14)	−0.0034 (10)	0.0008 (11)	0.0024 (10)
C22	0.0408 (12)	0.0492 (13)	0.0740 (15)	0.0024 (10)	−0.0011 (11)	0.0106 (12)
C23	0.0639 (17)	0.0627 (18)	0.121 (3)	−0.0079 (14)	0.0139 (17)	0.0197 (17)
C24	0.075 (2)	0.098 (3)	0.169 (4)	−0.008 (2)	0.039 (3)	0.050 (3)
C25	0.088 (3)	0.158 (4)	0.106 (3)	0.013 (3)	0.031 (2)	0.036 (3)
C26	0.090 (2)	0.155 (4)	0.086 (2)	−0.005 (3)	0.0237 (19)	−0.010 (2)
C27	0.0696 (18)	0.099 (2)	0.080 (2)	−0.0108 (16)	0.0145 (15)	−0.0080 (17)
C31	0.0422 (11)	0.0453 (12)	0.0592 (13)	−0.0070 (9)	−0.0006 (9)	0.0041 (10)
C41	0.0402 (11)	0.0480 (13)	0.0514 (12)	−0.0070 (10)	0.0076 (9)	−0.0033 (10)
C42	0.0614 (15)	0.0544 (15)	0.0585 (14)	−0.0130 (12)	0.0016 (11)	−0.0057 (11)
C43	0.0762 (17)	0.0521 (15)	0.0720 (17)	−0.0152 (13)	0.0109 (14)	−0.0111 (12)
C44	0.0662 (16)	0.0472 (14)	0.0708 (16)	−0.0058 (12)	0.0201 (13)	0.0017 (11)
C45	0.0647 (15)	0.0572 (15)	0.0528 (13)	0.0004 (12)	0.0119 (11)	0.0033 (11)
C46	0.0516 (13)	0.0492 (13)	0.0517 (13)	−0.0041 (10)	0.0064 (10)	−0.0045 (10)
C47	0.128 (3)	0.0530 (17)	0.098 (2)	−0.0051 (17)	0.030 (2)	0.0105 (15)
N1	0.0421 (10)	0.0507 (11)	0.0507 (10)	0.0011 (8)	−0.0007 (8)	0.0014 (8)
N2	0.0472 (12)	0.0668 (15)	0.0941 (17)	−0.0074 (10)	−0.0100 (11)	0.0156 (12)
N3	0.0435 (10)	0.0502 (11)	0.0685 (12)	−0.0082 (9)	−0.0078 (9)	0.0129 (9)

### Geometric parameters (Å, º)

**Table:**

C2—N1	1.342 (3)	C23—C24	1.404 (5)
C2—N3	1.349 (3)	C23—H23	0.9300
C2—C3	1.411 (3)	C24—C25	1.365 (6)
C3—C4	1.401 (3)	C24—H24	0.9300
C3—C31	1.420 (3)	C25—C26	1.341 (6)
C4—C5	1.390 (3)	C25—H25	0.9300
C4—C41	1.478 (3)	C26—C27	1.368 (4)
C5—C6	1.380 (3)	C26—H26	0.9300
C5—C9	1.501 (3)	C27—H27	0.9300
C6—N1	1.330 (3)	C31—N2	1.140 (3)
C6—C7	1.499 (3)	C41—C46	1.382 (3)
C7—C8	1.496 (4)	C41—C42	1.386 (3)
C7—H7A	0.9700	C42—C43	1.371 (4)
C7—H7B	0.9700	C42—H42	0.9300
C8—C9	1.531 (4)	C43—C44	1.384 (4)
C8—H8A	0.9700	C43—H43	0.9300
C8—H8B	0.9700	C44—C45	1.376 (3)
C9—H9A	0.9700	C44—C47	1.497 (4)
C9—H9B	0.9700	C45—C46	1.382 (3)
C21—N3	1.437 (3)	C45—H45	0.9300
C21—C22	1.494 (3)	C46—H46	0.9300
C21—H21A	0.9700	C47—H47A	0.9600
C21—H21B	0.9700	C47—H47B	0.9600
C22—C23	1.363 (4)	C47—H47C	0.9600
C22—C27	1.374 (4)	N3—H3	0.8600
			
N1—C2—N3	118.24 (19)	C22—C23—H23	119.9
N1—C2—C3	121.54 (18)	C24—C23—H23	119.9
N3—C2—C3	120.22 (18)	C25—C24—C23	119.3 (3)
C4—C3—C2	121.10 (19)	C25—C24—H24	120.3
C4—C3—C31	121.52 (19)	C23—C24—H24	120.3
C2—C3—C31	117.30 (18)	C26—C25—C24	120.7 (4)
C5—C4—C3	116.08 (19)	C26—C25—H25	119.7
C5—C4—C41	123.40 (19)	C24—C25—H25	119.7
C3—C4—C41	120.50 (18)	C25—C26—C27	119.7 (4)
C6—C5—C4	118.73 (19)	C25—C26—H26	120.2
C6—C5—C9	110.2 (2)	C27—C26—H26	120.2
C4—C5—C9	131.1 (2)	C26—C27—C22	122.0 (3)
N1—C6—C5	126.2 (2)	C26—C27—H27	119.0
N1—C6—C7	122.5 (2)	C22—C27—H27	119.0
C5—C6—C7	111.3 (2)	N2—C31—C3	174.7 (2)
C8—C7—C6	103.1 (2)	C46—C41—C42	117.6 (2)
C8—C7—H7A	111.1	C46—C41—C4	121.51 (19)
C6—C7—H7A	111.1	C42—C41—C4	120.9 (2)
C8—C7—H7B	111.1	C43—C42—C41	121.3 (2)
C6—C7—H7B	111.1	C43—C42—H42	119.4
H7A—C7—H7B	109.1	C41—C42—H42	119.4
C7—C8—C9	107.4 (2)	C42—C43—C44	121.4 (2)
C7—C8—H8A	110.2	C42—C43—H43	119.3
C9—C8—H8A	110.2	C44—C43—H43	119.3
C7—C8—H8B	110.2	C45—C44—C43	117.4 (2)
C9—C8—H8B	110.2	C45—C44—C47	121.1 (3)
H8A—C8—H8B	108.5	C43—C44—C47	121.5 (2)
C5—C9—C8	102.8 (2)	C44—C45—C46	121.6 (2)
C5—C9—H9A	111.2	C44—C45—H45	119.2
C8—C9—H9A	111.2	C46—C45—H45	119.2
C5—C9—H9B	111.2	C41—C46—C45	120.8 (2)
C8—C9—H9B	111.2	C41—C46—H46	119.6
H9A—C9—H9B	109.1	C45—C46—H46	119.6
N3—C21—C22	113.74 (19)	C44—C47—H47A	109.5
N3—C21—H21A	108.8	C44—C47—H47B	109.5
C22—C21—H21A	108.8	H47A—C47—H47B	109.5
N3—C21—H21B	108.8	C44—C47—H47C	109.5
C22—C21—H21B	108.8	H47A—C47—H47C	109.5
H21A—C21—H21B	107.7	H47B—C47—H47C	109.5
C23—C22—C27	117.9 (3)	C6—N1—C2	116.30 (19)
C23—C22—C21	121.4 (3)	C2—N3—C21	125.59 (19)
C27—C22—C21	120.7 (2)	C2—N3—H3	117.2
C22—C23—C24	120.3 (3)	C21—N3—H3	117.2
			
N1—C2—C3—C4	1.9 (3)	C23—C24—C25—C26	1.6 (6)
N3—C2—C3—C4	−178.91 (19)	C24—C25—C26—C27	−2.2 (6)
N1—C2—C3—C31	−174.95 (19)	C25—C26—C27—C22	0.6 (6)
N3—C2—C3—C31	4.2 (3)	C23—C22—C27—C26	1.6 (4)
C2—C3—C4—C5	−0.8 (3)	C21—C22—C27—C26	−176.9 (3)
C31—C3—C4—C5	175.9 (2)	C5—C4—C41—C46	134.4 (2)
C2—C3—C4—C41	−179.58 (18)	C3—C4—C41—C46	−46.9 (3)
C31—C3—C4—C41	−2.9 (3)	C5—C4—C41—C42	−47.7 (3)
C3—C4—C5—C6	0.1 (3)	C3—C4—C41—C42	131.0 (2)
C41—C4—C5—C6	178.90 (19)	C46—C41—C42—C43	1.5 (3)
C3—C4—C5—C9	−179.6 (2)	C4—C41—C42—C43	−176.5 (2)
C41—C4—C5—C9	−0.9 (4)	C41—C42—C43—C44	−1.7 (4)
C4—C5—C6—N1	−0.6 (3)	C42—C43—C44—C45	0.8 (4)
C9—C5—C6—N1	179.2 (2)	C42—C43—C44—C47	179.8 (3)
C4—C5—C6—C7	−179.6 (2)	C43—C44—C45—C46	0.4 (4)
C9—C5—C6—C7	0.2 (3)	C47—C44—C45—C46	−178.7 (2)
N1—C6—C7—C8	166.9 (2)	C42—C41—C46—C45	−0.4 (3)
C5—C6—C7—C8	−14.1 (3)	C4—C41—C46—C45	177.59 (19)
C6—C7—C8—C9	22.1 (3)	C44—C45—C46—C41	−0.6 (4)
C6—C5—C9—C8	13.4 (3)	C5—C6—N1—C2	1.6 (3)
C4—C5—C9—C8	−166.8 (2)	C7—C6—N1—C2	−179.4 (2)
C7—C8—C9—C5	−22.0 (3)	N3—C2—N1—C6	178.59 (19)
N3—C21—C22—C23	134.3 (2)	C3—C2—N1—C6	−2.2 (3)
N3—C21—C22—C27	−47.2 (3)	N1—C2—N3—C21	2.6 (3)
C27—C22—C23—C24	−2.2 (4)	C3—C2—N3—C21	−176.6 (2)
C21—C22—C23—C24	176.3 (3)	C22—C21—N3—C2	101.2 (3)
C22—C23—C24—C25	0.7 (5)		

### Hydrogen-bond geometry (Å, º)

Cg1 is the centroid of the N1/C2–C6 pyridine ring.

**Table:**

*D*—H···*A*	*D*—H	H···*A*	*D*···*A*	*D*—H···*A*
N3—H3···N2^i^	0.86	2.25	2.982 (3)	144
C47—H47*A*···*Cg*1^ii^	0.96	2.84	3.681 (4)	147

Symmetry codes: (i) −*x*, −*y*, −*z*; (ii) *x*, −*y*−1/2, *z*−3/2.
